# Which side do you take? Instruction versus action in resolving frame of reference conflicts

**DOI:** 10.3758/s13414-026-03239-2

**Published:** 2026-04-10

**Authors:** Christian Böffel, Matthias Joseph Becker, Jochen Müsseler

**Affiliations:** Institute of Psychology, Work and Engineering Psychology, RWTH Aachen University, Kackertstr. 9, 52072 Aachen, Germany

**Keywords:** Action effects, Avatar, Bottom-up, Simon effect, Stimulus-response compatibility, Top-down

## Abstract

Previous studies of human-avatar interaction have shown that users are able to take the perspective of an avatar and act as if they were in the avatar’s place. As a result, the positions of objects are encoded from the avatar’s perspective and within the avatar’s spatial reference frame, and avatar-based compatibility effects can be observed. In the present study, we confronted participants simultaneously with two different avatars that offered conflicting visual perspectives. We asked participants to take the perspective of one avatar while performing a Simon task. In one half of the experiment, participants controlled the hands of the instructed avatar, while in the other half, they controlled the opposite avatar. We used stimulus-response compatibility effects to examine which perspective participants took, allowing us to examine whether instruction or control was more important in resolving frame of reference conflicts. Overall, we observed a compatibility effect based on the position of the controlled avatar, even when the participants were instructed to take the perspective of the opposite avatar. This suggests that the stimuli were encoded from the perspective of the controlled avatar, despite receiving conflicting instructions. The influence of action control overpowered the top-down influence of the instruction.

## Introduction

Avatars are used in a growing number of digital applications to move through and interact with virtual worlds. They serve as a link between the digital and analog worlds by translating a user’s (physical) input into virtual actions and by providing a spatial frame of reference in which events and actions are encoded (cf. Dolk et al., 2013; Müsseler et al., 2022). Coordinating between user and avatar is required to ensure that these translations occur optimally. One means of coordination is spatial perspective taking (Flavell, 1977). Traditionally, perspective taking (PT) has been studied as a social phenomenon. For example, previous research has shown that visual perspective taking is related to theory of mind, as whether the target of PT has visual access to an object or not can be relevant for PT (Freundlieb et al., 2017). However, imagining how a scene looks from another perspective is also possible when the target of PT is not a human, but an object (Quesque et al., 2020) or an avatar. This is particularly the case when agency is experienced from or through them (Böffel & Müsseler, 2019b; Zwickel, 2009).

A crucial aspect of experiencing agency is the consequence of one’s action. So-called action effects are self-produced events that cause feelings of agency (Weller et al., 2017). The link between an action and its effect can be established through experience (Elsner & Hommel, 2001), and extensive research has demonstrated this binding process and its importance for action planning in a variety of tasks and modalities. Examples include forced and free-choice responses (Pfeuffer et al., 2023), manual responses, or saccades (Gouret & Pfeuffer, 2023), for spatial or verbal-semantic overlap (Koch et al., 2021). The link between action and effect can be regarded within the Theory of Event Coding (TEC, Hommel et al., 2001), which incorporates the ideomotor principle in which actions are represented as their anticipated effects (Greenwald, 1970). Once the relationship between action and effect is sufficiently learned through experience, TEC proposes that actions are encoded as their sensory effects (Hommel, 2019). However, until the link between action and effect is sufficiently established, the required updating of the action’s feature codes can be viewed as a bottom-up process. Taken together, both bottom-up and top-down processes are relevant to the relationship between actions and their effects, with the importance of the top-down influence of the associated effect likely increasing over time with increasing experience.

Previous studies have shown that this action-effect association not only influences spontaneous visual PT but also enables PT in situations where it is typically not observed (Böffel & Müsseler, 2019b). When compatibility effects of controlled and independently acting avatars are contrasted, the compatibility effects for controlled avatars are larger (von Salm-Hoogstraeten & Müsseler, 2021). Additionally, when two avatars are presented simultaneously, with one being controlled while the other acts independently, the controlled avatar’s perspective is generally taken, underscoring the importance of control for the emergence of avatar-based compatibility effects (von Salm-Hoogstraeten & Müsseler, 2021).

While action effects play an important role, other influences on visual PT have also been observed, such as different instructions. Böffel and Müsseler (2018) managed to influence PT by providing different explanations for the same action effect. While both instructions and action effects have been shown to influence PT, it remains unclear which of the two is more influential overall and how their influence develops over the course of the human-avatar interaction. Is an instruction sufficient to achieve visual PT that is required to solve the task even if it is in conflict with the events that are experienced?

The present study therefore investigated such bottom-up and top-down influences on the spatial encoding of visual stimuli by introducing a conflict between an instruction and an action effect. The influence of the instruction can be seen as top-down as it arbitrarily focuses on one of two identical avatars. As participants were not informed about the action effect prior to the experiment, the association between action and effect had to be experienced first, and its influence can therefore be seen as bottom-up until the association is sufficiently learned. We used the paradigm of the avatar-Simon effect (Böffel & Müsseler, 2019a) but with two opposing avatars providing different and conflicting frames of reference. The setup is therefore similar to the second experiment by von Salm-Hoogstraeten and Müsseler (2021), but we instructed participants to focus on one avatar, and which avatar was controlled changed over the course of the experiment. Using the logic of stimulus-response compatibility, we determined whether instruction or experienced action control is more influential in taking the perspective of the avatars. Similar approaches have been used before to examine conflicting frames of reference (e.g., Baess et al., 2018), but instead of examining allocentric versus egocentric frames of reference, both relevant frames of reference in the present study are allocentric and designed to be equivalent.

In the current study, compatibility effects are defined from the controlled avatar’s point of view. This means that the more positive these observed compatibility effects are, the stronger the bottom-up influence of the action effect is. The conflict between top-down and bottom-up influences arises in conditions in which the participant is instructed to take the perspective of the avatar that is not controlled. Here, a negative compatibility effect would indicate that compatibility is determined by the instructed instead of the controlled avatar, while a positive compatibility effect would again indicate a stronger (bottom-up) influence of the action effect.

Based on the importance of action effects for human-avatar interaction and avatar perspective taking, we propose the following hypotheses: The location of the action effect will resolve a conflict of instruction versus effect in its favor. As a result, a stimulus-response compatibility effect (faster responses and fewer errors) will be observed from the perspective of the controlled instead of the instructed avatar (H1). However, we still expect to observe a significant influence of instruction, resulting in an interaction effect between instruction and compatibility, with larger compatibility effects when the controlled avatar is also instructed compared to the opposite avatar (H2).

## Method

### Participants and sensitivity

A total of 38 participants (17 female, 21 male) with a mean age of 24.2 (*SD* = 3.6) years participated in this experiment and were eligible to receive partial course credit for their participation. Participants were recruited using a university mailing list. All participants had normal or corrected-to-normal vision and provided informed consent for the terms of data collection, use, and storage.

The sample size was based on the original laboratory-based experiment by Böffel and Müsseler (2019b) of 24, which we increased to account for a potential decrease in measurement accuracy when conducting an online versus a laboratory study (cf. Böffel & Meinardus, 2024). The total sample size allowed for the detection of compatibility effects down to an effect size of *d* =.47 with a power of (1 – β) =.80, according to G*Power, estimated for two-tailed repeated-measures t-tests and α =.05 (Faul et al., 2007).

### Apparatus and stimuli

**Fig. 1:**
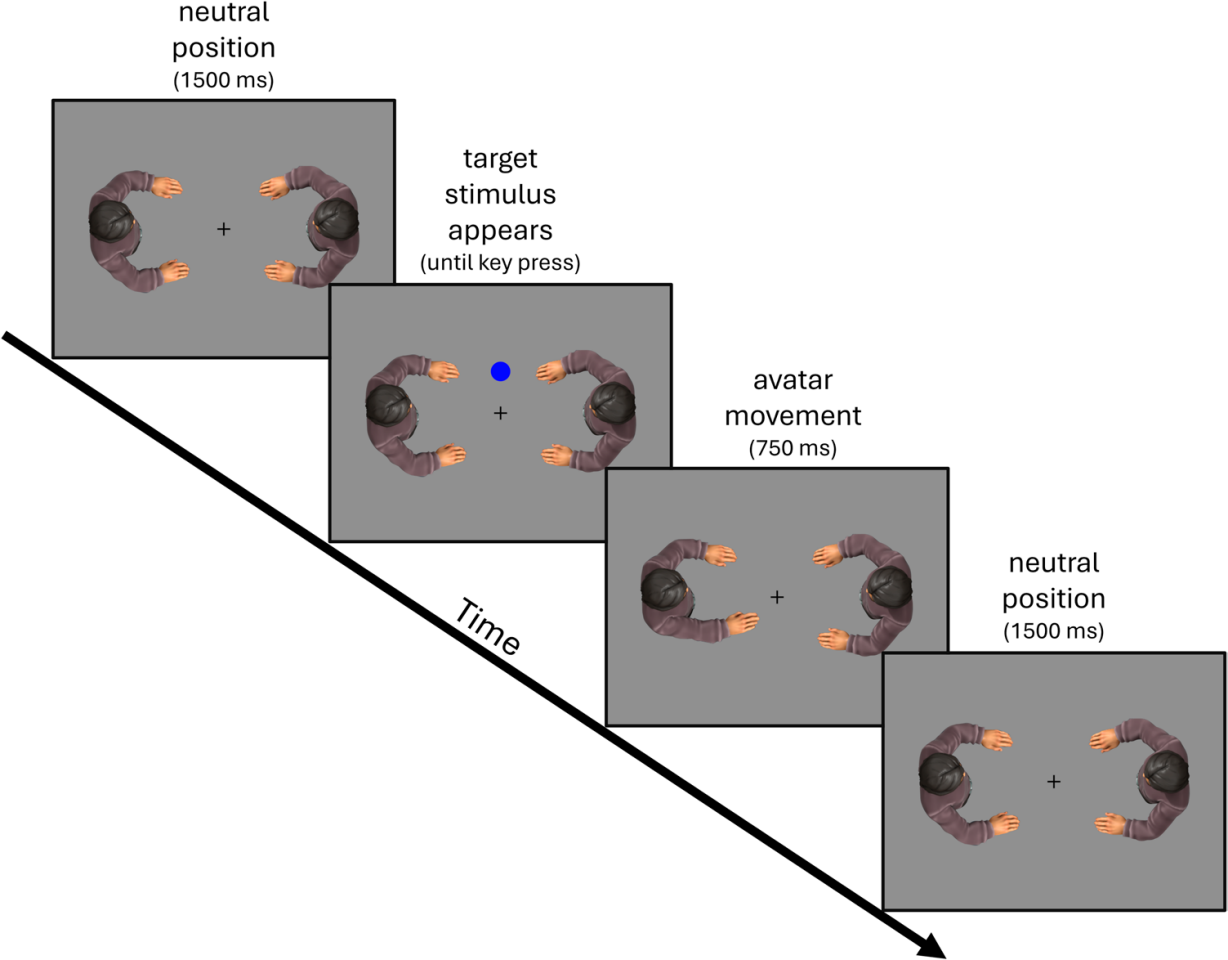
Examples of experimental conditions in which a participant controlled the left avatar but was instructed to take the perspective of the right one. Which avatar was controlled first and which avatar instructed was counterbalanced between participants. A dark blue target stimulus is shown at the top position. The target stimulus can be seen on the left from the perspective of the left avatar and on the right from the perspective of the right avatar. In this example, the left avatar is controlled. After the participant responded by pressing the right key, the left avatar moved its right hand slightly

The experiment was conducted online, using the browser-based version of Psytoolkit (Stoet, 2010, 2017), and participants accessed the experiment using their own computers at home. Light and dark blue disks screen (60 pixels in diameter) were presented as target stimuli in front of a gray background. They were located along a vertical axis in the center of the screen and could appear either above or below it. Similar to Böffel and Müsseler (2019b), avatars (approximately 305 × 370 pixels) were presented on the left and right side of the screen. However, instead of having only one avatar active at the same time, two identical avatars were shown facing each other in the screen center. A fixation cross was presented between avatars (Fig. 1).

### Procedure

Participants were first informed about the conditions of the study and data collection and gave their informed consent before participating. They were then asked for their age and gender before the actual experiment began. The participants were instructed to place their index fingers on the “X” and “M” keys and keep them there throughout the experiment. They were also told to carry out the experiment in a quiet environment and to focus entirely on the task, without doing anything else at the same time. Participants performed a basic Simon task and were instructed to respond to the color of the disks by pressing the left (“X”) or right (“M”) key on their keyboard. The mapping of color to key press was counterbalanced across participants. Both avatars and the fixation cross remained visible throughout the experiment. The participants were randomly assigned to one of two groups: Group 1 was instructed to take the perspective of the left avatar throughout the experiment and group 2 was instructed to take the perspective of the right avatar. The presentation of the target stimuli, i.e., the light or dark blue disk, at the top or bottom position marked the beginning of a trial and the reaction time was recorded when the participant pressed a key. After a key press, the controlled avatar moved the spatially corresponding hand in a single frame. The moved hand was displayed for 750 ms. If the response was incorrect, it was counted as an error and an additional error message was displayed. The experiment continued with the next trial after an intertrial interval of 1,500 ms.

The experiment was divided into two halves. Within each half, each combination of stimulus color (dark vs. light blue) and stimulus position (top vs. bottom) was repeated a total of 45 times, organized in sets of 12 trials containing three repetitions of each condition in a randomized order. The first set was considered practice trials and excluded from the analysis. In one half, participants controlled their instructed avatar; in the other half, they controlled the opposing avatar. The order was counterbalanced across participants. In total, each participant completed 360 trials (including 24 practice trials) and took approximately 20 min to complete the experiment.

### Design

In accordance with the main hypothesis, stimulus-response compatibility was defined based on the perspective of the controlled avatar. If a stimulus appeared to the right of the controlled avatar, a right key press was considered compatible and a left key press was considered incompatible. If a stimulus appeared to the left of the controlled avatar, a left key press was considered compatible and a right key press was considered incompatible. This resulted in a 2 × 2 design with the within-subjects factors compatibility (incompatible vs. compatible) and control (instructed avatar is controlled vs. opposite avatar is controlled).

## Results

Reaction times (RTs) longer than 1,000 ms (300 trials, 2.35%) were excluded from the analyses. No anticipations, i.e., RTs shorter than 100 ms, were observed. In addition, a total of 375 errors (2.94%) were excluded from the RT analysis. Mean RTs and percentage errors (PEs) were analyzed separately using 2 × 2 within-subjects ANOVAs with the factors *compatibility* (incompatible vs. compatible from the perspective of the controlled avatar) and *control* (instructed avatar is controlled vs. opposite avatar is controlled).

### Reaction times

**Fig. 2:**
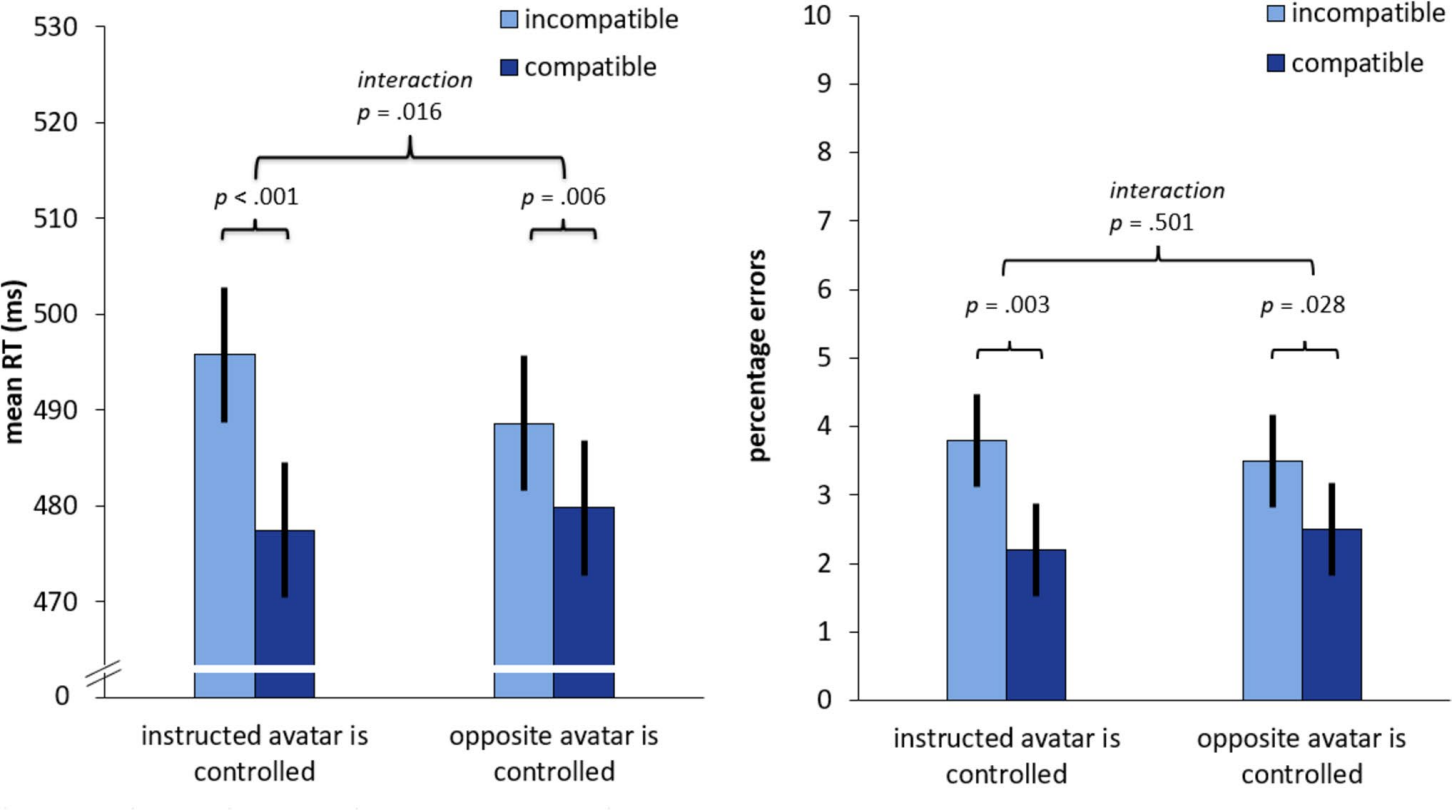
Mean Reaction times (RTs) and error rates as a function of compatibility from the controlled avatar’s perspective and avatar rotation. Error bars represent 95% within-subject CIs (Loftus & Masson, 1994)

Means of RTs and error rates per condition are shown in Fig. 2. A main effect of *compatibility* was observed, *F*(1, 37) = 32.902, *p* <.001, η_p_^2^ =.471. Responses were faster in compatible compared to incompatible conditions (*M*_incompatible_ = 492 ms, *M*_compatible_ = 479 ms). The factors *compatibility* and *control* significantly interacted, *F*(1, 37) = 6.426, *p* =.016, η_p_^2^ =.148. When the instructed avatar was controlled, compatible conditions were associated with faster responses compared to incompatible conditions (*M*_incompatible_ = 496 ms, *M*_compatible_ = 477 ms). Pairwise t-tests showed that this compatibility effect was significant, *t*(37) = 6.154, *p* <.001. This compatibility effect (i.e., the difference between incompatible and compatible conditions) was smaller in conditions in which the avatar opposite to the instructed avatar was controlled (*M*_incompatible_ = 489 ms, *M*_compatible_ = 480 ms). This compatibility effect was also significant, *t*(37)= 2.900, *p* =.006. No significant main effect of *control* was observed, *F*(1, 37) =.214; *p* =.647, η_p_^2^ =.006.

### Percentage errors

The analysis of percentage errors revealed a significant main effect of *compatibility*, *F*(1, 37) = 19.925, *p* <.001, η_p_^2^ =.350. Fewer errors were observed in compatible compared to incompatible conditions (*M*_incompatible_ = 3.7%, *M*_compatible_ = 2.3% ms). No significant main effect of *control* (*F*(1, 37) =.009, *p* =.923, η_p_^2^ <.001) or significant interaction between both factors were observed (*F*(1, 37) =.461, *p* =.501, η_p_^2^ =.012).

### Exploratory analyses

#### Analysis split by avatar, stimulus, and response position

We conducted two additional mixed ANOVAs, one for mean RTs and one for PEs to examine the participants’ behavioral patterns in greater detail. These analyses allow for comparison with previous studies that used similar analyses, for example, with regard to potential spatial asymmetries of the compatibility effect. However, this analysis is slightly limited in the present study, because the position of the instructed avatar was fixed for each participant. We included the within-subjects factors *position of the controlled avatar* (right vs. left); *stimulus position* (top vs. bottom) and response position (left vs. right key press) in the analyses. We additionally added the *instructed avatar position* (left vs. right) as a between-subjects factor in the analysis, resulting in two 2 × 2 × 2 × 2 mixed ANOVAs.

For mean RTs, only the three-way interaction of *controlled avatar position*, *stimulus position*, and *response position* was significant, *F*(1, 36) = 30.392, *p* <.001, η_p_^2^ =.458. This interaction is aligned with the observed main effect of compatibility after collapsing these factors in the main analysis. All other effects were not significant.

For mean percentage errors, only the three-way interaction of *controlled avatar position*, *stimulus position*, and *response position* was significant, *F*(1, 36) = 18.863, *p* <.001, η_p_^2^ =.344, again aligning with the observed main effect of compatibility with a combined compatibility factor. No other significant effects were observed.

#### Temporal dynamics of compatibility effects

We conducted an exploratory analysis to investigate how compatibility effects change over the course of the experiment. There are two possible mechanisms that could cause a change of the observed compatibility effects over the course of the experiment. First, the instruction to take the perspective of an avatar was given only at the beginning of the experiment. Since no additional reminder is provided, the influence of this instruction may have faded over the course of the experiment. At the same time, the association between action and effect is learned by observing the consequences of the performed key presses, so it is expected that the influence of the action effect would increase over time.

**Fig. 3:**
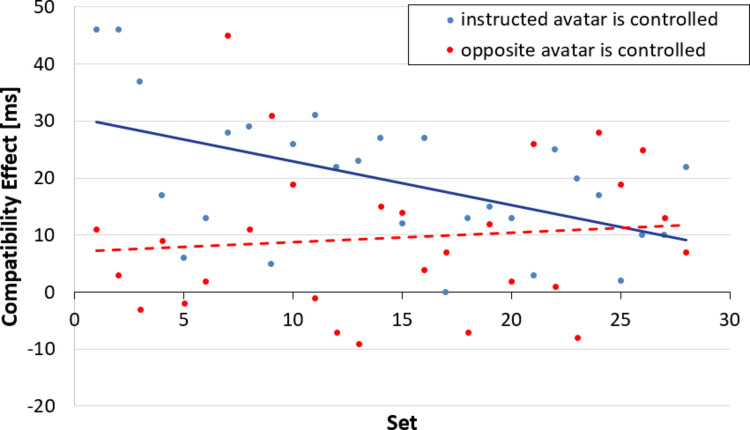
Compatibility effects over the course of the experiment. Compatibility effects were defined from the controlled avatar’s perspective. Each set contained 12 trials

To examine the size of the compatibility effects over the course of the experiment, the trials were organized by trial number into sets of 12 trials, i.e., the first set contained the first 12 trials, and set 28 contained the last 12 trials of the experiment. Sets of 12 were chosen because the design of the experiment ensured an equal number of trials of each condition within each set, which ensured that compatibility effects could be properly calculated for each participant. Mean compatibility effects were then calculated for each condition of avatar control (instructed avatar controlled vs. opposite avatar controlled) and set, and the Pearson correlation between set number and mean compatibility effect was calculated and is visualized in Fig. 3.

If the effect of instruction diminished over the course of the experiment, a negative correlation would be expected when the instructed avatar was controlled, and a positive correlation when the opposite avatar was controlled. On the other hand, if the association between action and effect strengthened over the course of the experiment, a positive correlation between set number and compatibility effect would be expected in both conditions. We ran the correlation analysis across the entire experiment because the instruction about which avatar’s perceptive to take was given only once at the beginning of the experiment. Therefore, the set number accurately reflects the time that has elapsed since this instruction was given. Additionally, further analyses of a possible order effect, i.e., whether the instructed or other avatar was controlled first, revealed no significant effects of the order factor. We found a significant, negative correlation when the instructed avatar was controlled, *r*(26)_instructed___controlled_ = -.518, *p* =.005, but no significant correlation when the opposite avatar was controlled, *r*(26)_opposite___controlled_ =.105, *p* =.595.

## Discussion

This study investigated whether instruction or action control is more important in resolving the conflict between two reference frames provided by avatars. In contrast to previous studies, which generally investigated conflict between egocentric and allocentric reference frames, our study focused on the conflict between two allocentric reference frames and how this conflict is resolved. Overall, we observed a compatibility effect defined from the perspective of the controlled avatar, indicating that action control was more important overall. It also constitutes evidence for an avatar-Simon effect, indicating that stimuli were regarded within the controlled avatar’s frame of reference (Böffel & Müsseler, 2019b). However, we also found evidence for the influence of the instruction to take the perspective of one of the avatars. From the observed interaction of compatibility and instruction, it appears that instructing a person to take the perspective of the uncontrolled avatar led to a significantly smaller compatibility effect from the perspective of the controlled avatar compared to the scenario in which a person was instructed to take the perspective of the controlled avatar. However, even in the case of a conflict between control and instruction, a significant compatibility effect was observed in favor of the controlled avatar, in terms of both mean RTs and percentage errors. Our results highlight that experienced control over the avatar shapes perspective taking and they underline the importance of action effects in line with previous studies (Böffel & Müsseler, 2019a, 2019b; for an overview, see Müsseler et al., 2022).

Furthermore, the results also suggest that how the participants’ actions were encoded was influenced by their associated effects, as stated by the ideomotor principle (Greenwald, 1970) and the TEC (Hommel et al., 2001). From their own point of view, the participants were pressing the left or right key in response to stimuli presented at the top or bottom of the screen. In the current study, two opposing avatars meant that the same stimuli could be perceived as being on either the left or the right, depending on which avatar’s perspective is taken. The results showed that this conflict was resolved through control of the avatar, i.e., the observed effects of the action on the screen. The connection between the pressing of a key and the subsequent hand movement on the screen is learnt and anticipated in subsequent trials, including the avatar that will perform the action. Once this occurs, the spatial coding of subsequently presented target stimuli is also based on this anticipation of the controlled avatar and the action effect.

The exploratory analyses investigating the interaction between avatar positions, response position, and stimulus position revealed no additional significant effects. Unlike previous studies, no significant asymmetries based on response positions or avatar positions were observed (cf. Böffel & Müsseler, 2020). Furthermore, no evidence of an egocentric (orthogonal) compatibility effect from the participants’ point of view was found, as the stimulus position and response position factors did not interact significantly. This observation is in line with previous studies (e.g., Böffel & Müsseler, 2019b) and suggests that the presence of avatars overrode any potential compatibility effects, since the avatars’ frame of reference takes precedence.

The correlation analysis over the course of the experiment suggests that the effect of the instruction is likely to diminish over time. This seems plausible because the instruction is only presented once at the beginning of the experiment, whereas the action effect is constantly re-experienced by the participants. In the case of conflict between control and instruction, Fig. 3 suggests that this conflict is likely already resolved and that the action-effect relationship already established during practice trials. This demonstrates the flexibility of the mental representation of actions and how quickly it is shaped by observing the associated effects.

## Implications and future research

The need to consider different perspectives and frames of reference is not limited to the digital world and the human-avatar interaction. It is also crucial for certain tasks in the work environment, and future research could investigate conflicting frames of reference and their resolution in real-world settings, for example in collaborative tasks (cf. Galati & Avraamides, 2013). Furthermore, the mechanisms of frame of reference conflict resolution should be further investigated, for example by focusing on the role of attention. It seems likely that two conflicting frames of reference also compete for a person’s attention. Recent studies (e.g., Ford et al., 2024) have demonstrated the role of attentional shifts for visual PT and other properties of the situation could also lead to attentional shifts that might in turn cause a conflict resolution. Another interesting question relates to the potential role of an egocentric frame of reference. While in the current task the conflicting frames of reference are both allocentric, another interesting question is whether the controlled avatar is perceived as an extension of the person’s self (cf. Müsseler et al., 2022), allowing one frame of reference to be re-coded as egocentric, effectively re-establishing an egocentric frame of reference.

## Conclusion

Overall, the results highlight the importance of action control in human-avatar interaction. An avatar that is actually controlled by the person serves as a proxy for their actions and is therefore crucial for planning further actions through this avatar, for example to interact with the virtual world shown on the screen. This avatar automatically becomes “theirs” and overrides any possible effects of instruction. If the goal is to take the perspective of a particular avatar, then the action effects and the experience of controlling that avatar are critical to achieving that goal.

## Data Availability

Data and materials for this study are available via the following link: https://osf.io/cskx2/?view_only=b61097f7b4254662aa4d28203675c27d.

## Code availability

The Psytoolkit-Syntax for this study is available via the following link: https://osf.io/cskx2/?view_only=b61097f7b4254662aa4d28203675c27d.

Other code is available from the corresponding author upon request.

## Declarations

### Open practices statement

Data and materials for the study are available via the following link: https://osf.io/cskx2/?view_only=b61097f7b4254662aa4d28203675c27d

The experiment was not preregistered.
